# Genetic Background Modulates the Phenotype of a Mouse Model of DYT1 Dystonia

**DOI:** 10.1371/journal.pone.0032245

**Published:** 2012-02-29

**Authors:** Lauren M. Tanabe, Caitlin Martin, William T. Dauer

**Affiliations:** 1 Department of Neurology, University of Michigan Medical School, Ann Arbor, Michigan, United States of America; 2 Emory University School of Medicine, Atlanta, Georgia, United States of America; 3 Department of Cell and Developmental Biology, University of Michigan Medical School, Ann Arbor, Michigan, United States of America; University of California Los Angeles, United States of America

## Abstract

DYT1 dystonia is a debilitating neurological disease characterized by involuntary twisting movements. The disease is caused by an in-frame deletion (GAG, “ΔE”) mutation in the TOR1A gene that encodes the torsinA protein. Intriguingly, only 30% of mutation carriers exhibit motor symptoms despite the fact that functional brain imaging studies show abnormal brain metabolism in all carriers. Because genetic modifiers may be a determinant of this reduced penetrance, we examined the genetic contribution of three different inbred strains of mice on the DYT1 mutation in animals that are homozygous (*Tor1a^ΔE/ΔE^*) or heterozygous (*Tor1a^ΔE/+^*; disease state) for the disease-causing ΔE mutation. We find that the DBA/2J, C57BL/6J, and CD1-ICR contribution of genes significantly alter lifespan in *Tor1a^ΔE/ΔE^* mice, which die during the first few days of life on the 129S6/SvEvTac (129) background. The C57BL/6J (B6) strain significantly decreases life expectancy of *Tor1a^ΔE/ΔE^* animals but, like 129S6/SvEvTac *Tor1a^ΔE/+^* mice, congenic C57BL/6J *Tor1a^ΔE/+^* mice do not exhibit any motor abnormalities. In contrast, the DBA/2J (D2) strain significantly increases life expectancy. This effect was not present in congenic DBA/2J *Tor1a^ΔE/ΔE^* mice, indicating that the extended lifespan of F2 129/D2 mice was due to a combination of homozygous and heterozygous allelic effects. Our observations suggest that genetic modifiers may alter the penetrance of the ΔE mutation, and that mapping these modifiers may provide fresh insight into the torsinA molecular pathway.

## Introduction

Dystonia is defined as abnormal involuntary movements that are prolonged, twisting in nature and frequently stereotypic and repetitive. Dystonia occurs as an isolated symptom without evidence of brain injury (“primary” dystonia) or as a consequence of pathologic insults to the basal ganglia or related structures (“secondary” dystonia). Primary and secondary dystonia may be treated with similar medications (e.g. anticholinergics) and both respond to deep brain stimulation therapy [1]. These facts, and the characteristic dystonic postures that result from diverse etiologies, suggest that primary and secondary forms of dystonia may share a common downstream abnormality, perhaps a stereotyped disruption of basal ganglia output (from the internal segment of the globus pallidus/substantia nigra pars reticulata).

The most common genetic form of primary dystonia, DYT1 dystonia, is a neurodevelopmental disorder caused by an in-frame deletion (GAG, “ΔE”) in the TOR1A gene that results in the loss of a glutamic acid in the C-terminus of torsinA [2], [3]. DYT1 dystonia is dominantly inherited but abnormal movements affect only 30% of mutation carriers. Despite this incomplete penetrance, 2-deoxyglucose studies show that all carriers exhibit abnormal brain metabolism, with increased metabolic activity in the cerebellum, putamen/globus pallidus, and supplementary motor cortex [4], [5]. Similarly, magnetic resonance diffusion tensor imaging (DTI) shows white matter abnormalities associated with reduced integrity of the cerebellothalamocortical motor pathway in all DYT1 mutation carriers [6], [7]. These clinical studies highlight that the apparent penetrance of a mutation depends greatly on the phenotype being assessed, and demonstrate that all ΔE mutation carriers have abnormally functioning brains.

The factors that determine conversion from sub-clinical “endophenotype” to overt disease remain unknown. Similarly, nearly all animals harboring monogenic mutations show significant phenotypic variability, likely due to multiple intermingling factors such as environment, allelic heterogeneity and stochastic effects, as well as the presence of modifier genes [8]. Indeed, a focus on the effect of this “genetic background noise” [8] is emerging in an effort to understand what makes some individuals more susceptible than others to certain disease-causing mutations. The features of DYT1 dystonia (monogenic mutation, incomplete penetrance) suggest that this disease may be an excellent model system in which to examine these issues. Possible genetic modifiers of the torsinA pathway include torsinB, which has redundant functions [9], and other torsinA-interacting proteins, including LAP1, LULL1 [10] and printor [11]. Importantly, identifying factors that modulate ΔE-torsinA phenotypes has the potential not only to provide insight into disease mechanism, but also may suggest alternative strategies for disease treatment and prevention.

Given the many factors that can modulate disease phenotypes, it can be exceedingly difficult to model diseases with limited penetrance, such as DYT1 dystonia. To date, etiologic mouse models of DYT1 dystonia do not have any obvious dystonic features or evidence of pathology such as neuronal loss, including transgenic mice expressing human mutant torsinA (hMT) [12], [13], [14], and heterozygous knock-in mice in which the ΔGAG mutation has been introduced in the endogenous mouse *Tor1a* gene [15], [16]. Furthermore, homozygous mutant torsinA expression results in perinatal lethality [17] preventing behavioral analysis of these mice. Therefore, mouse models of DYT1 dystonia suffer from an “all or none” effect of mutated torsinA in mice. We set out to explore ways to: 1. Amplify any behavioral abnormalities in the disease state mouse (heterozygous) or 2. Temper the effects of homozygous *Tor1a^ΔE/ΔE^* mouse (increase lifespan to observe effects).

The lack of a consistent or clearly apparent phenotype may be due in part to the variability in mouse backgrounds used in these studies. Modifier genes present in certain strains may act to suppress or exacerbate the effects of the ΔE mutation. Numerous studies demonstrate that genetic background alters both baseline and pharmacological responses in mice [18]. Modifier loci have already been mapped for several neurological diseases in both human and mouse. These diseases include tremor, dystonia, epilepsy and Huntington's disease. For example, in the kinesiogenic mouse model of dystonia, *Scn8a^medj^* mice exhibit striking phenotypes on the C57BL/6J background with paralysis and lethality by one month of age, while the C3H inbred strain background causes a progressive dystonia and ataxia, but a normal lifespan [19]. To begin to identify genetic modifiers of the torsinA pathway, we utilized mice homozygous for the ΔE mutation (*Tor1a^ΔE/ΔE^*). These mice, which die on the day of birth [16], have histologically normal-appearing brains, but electron microscopic (EM) analysis shows a selective disruption of the neuronal nuclear envelope (NE; referred to as “blebs”) [16]. We used these phenotypes (animal death, NE blebs) as an *in vivo* read-out of torsinA function, and explored whether they were modified when placed on distinct genetic backgrounds, a strategy similar to how the rough eye phenotype is utilized in drosophila genetic studies. We pursued an F1 intercross screening strategy to identify background strains that amplify or suppress death or NE blebs. Subsequently, we generated congenic mice on these different genetic backgrounds to test for an effect of background on the behavioral phenotype of *Tor1a^ΔE/+^* mice. We find that despite the effect of C57BL/6J alleles on the survival of *Tor1a^ΔE/ΔE^* mice, these alleles did not cause a behavioral phenotype in the congenic *Tor1a^ΔE/+^* mice (disease genotype).

## Materials and Methods

Male mice were housed in groups of 5 and maintained on a 12-hour light/dark schedule (lights on at 7:00 pm). Food and water were provided *ad libitum*. Behavioral testing occurred during the dark phase between 7:00 am and 5:00 pm. Animal testing was conducted in accord with the National Institutes of Health laboratory animal care guidelines and with the University Committee on Use and Care of Animals at the University of Michigan approval. The University of Michigan's Institutional Unit for Laboratory Animal Medicine (ULAM) provides veterinary care to all animals used on campus. We ensured that all animals used in this study were healthy and experienced minimal discomfort. All protocols were approved prior to experimentation. Specifically, experiments were described in protocol 10292.

### Generation of Tor1a^ΔE/+^ congenic mouse strains and intercross breeding strategy

The *Tor1a^ΔE/+^*mouse, with a targeted deletion of glutamic acid (ΔE) in the encoded protein torsinA, was generated as previously described [16] by gene targeting in ES cells from the 129S6/SvEvTac (129) strain. Heterozygous 129-*Tor1a^ΔE/+^* mice were mated to C57BL/6J (B6), DBA/2J (D2), and CD1-ICR (CD1) mice to initiate 3 lines of *Tor1a^ΔE/+^* mice on different genetic backgrounds. F1 mice were intercrossed to generate F2 *Tor1a^ΔE/ΔE^* mice with genetic backgrounds that were ∼50% of the original 129 background and ∼50% of B6, D2, or CD1 background (referred to as 129/B6, 129/D2, and 129/CD1, respectively).

To generate congenic B6·*Tor1a^ΔE/+^* mice, 129-*Tor1a^ΔE/+^* heterozygotes were repeatedly backcrossed to the B6 and D2 strains for more than 10 generations to generate two different strains with 99.6–99.8% genetic identity with the B6 and D2 inbred strains.

### Genotyping

Tail samples from mice were excised and boiled in 300 µl 50 mM NaOH at 95°C for 50 minutes. Denatured tails were vortexed and mixed with 30 µl 1 M Tris pH 8.0 buffer to neutralize and centrifuged for 10 minutes at maximum speed. Pre-mixed PCR beads (PuReTaq Ready-To-Go PCR Beads, GE healthcare) were resuspended in 24 µl of primer mix at final concentration of 3.0 µg/ml and 1.0 µl of tail lysate supernatant. Primer sequences and PCR parameters for genotyping are listed in Table 1.

**Table 1:** Genotyping parameters for *Tor1a* knock-in mice.

Mutant animal	Primers	PCR parameters	Product size
*Tor1a^ΔE/+^*	Forward: 5′-agtctgtggctggctctccc-3′	95°C for 1 min	WT = 300 bp
	Reverse: 5′- cctcaggctgctcacaaccac-3′	95°C for 15 sec	Mut = 340 bp
		68°C for 30 sec	
		72°C for 30 sec	
		Repeat 38 times	
		72°C for 10 min	

### Sequencing genomic DNA

For sequencing of the *Tor1a* mouse gene, DNA was extracted and purified from mouse tail samples using Qiagen DNeasy Blood and Tissue kit per manufacturer's instructions. Sequencing was done using an ABI Model 3730 sequencer with the following primers: 5′- AAC AGA GCC AAC ACT CTG G-3′ (forward) and 5′-TGC TGT ACA AGA TCC TCC-3′.

### Behavioral Studies

All mice were kept on a reverse light dark cycle. Behavioral tests were performed during the dark period when animals were most alert. Independent cohorts were used for the baseline open-field and drug challenge open-field studies. Only male mice were used for behavioral studies.

#### Open-field test

The open-field test was used to assess animals' locomotor activity. Animals (*n* = 9–15 for each genotype) were placed in one of the five square open field boxes 43×43 cm^2^ with two sets of 16 pulse-modulated infrared photobeams (MED Associates) that records the animal's location and path (horizontal activity), as well as the number of rears (vertical activity) located inside sound-attenuating cabinets with fans. Illumination of the test room was the same as the mouse colony room. Mice were examined at 6 months, 9 months and 12 months of age. For baseline experiments, mice were placed in the open-field chamber for 60 minutes. Data were analyzed as distance traveled (cm) and rearing in 5-minute bins over time.

#### Rotarod

Rotarod (Ugo Basile, model 47600) was used to assess the mouse's ability to maintain balance and coordination (*n* = 10–15 for each genotype). The apparatus consists of five 3 cm diameter drums with six flanges dividing the drum, accommodating up to five mice. Mice were placed in one of the five allocated slots on the rotarod and latency to fall was measured. There were two components to this test – training and challenge. Rotarod training occurred over 3 consecutive days. For training, animals were placed on the rotarod as it accelerated from 4 rpm to 40 rpm over 6 minutes. The trial ended when the mice either fell off the rod or 400 seconds elapsed. Four trials were performed on each of the three days. On the testing day, mice were placed on the rotarod at 3 different fixed speeds, 4 trials per speed, for a total of 12 trials. On this day, the trial ended when the mouse fell off the rod or 300 seconds elapsed.

#### Balance Beam

Mice were trained to cross a square 80 cm long sanded plastic beam 5 mm wide, which was elevated 50 cm above base level (*n* = 9–14 for each genotype). At the start of each trial, mice were placed on clear open platform. A dark box at the opposite end of the beam provided motivation for the mouse to cross the beam. Traversal time and number of foot slips were measured as mice traversed the beam. Mice were tested daily with two trials on four consecutive days. The protocol was adapted from Shokkattai and colleagues [20].

### Drug Treatment

Three drugs dissolved in saline were administered by i.p injection – scopolamine, GBR1290 and quinpirole. All drugs were purchased from Sigma. Animals' locomotor and rearing activities were monitored following a 30-minute habituation period and immediately after drug injection for the locomotor activating drugs: scopolamine (1.0 mg/kg, i.p.; *n* = 10–14 for each genotype receiving drug) and GBR12909 (5.0 mg/kg, i.p.; *n* = 5–8 for each genotype receiving drug and 4–7 for each genotype receiving vehicle). For the locomotor-depressing quinpirole (0.1 mg/kg, i.p.; *n* = 10–11 for each genotype receiving drug and 6–7 for each genotype receiving vehicle), there was no habituation period prior to injection and activity was monitored immediately after the challenge.

### Data Analysis

The effects of strain on lifespan of *Tor1a^ΔE/ΔE^* mice were analyzed with survival curves using the Kaplan and Meier method created with GraphPad Prism 4.0. Two or more survival curves were compared using the logrank test, and Chi-square test was used to generate p values.

Behavioral data were subject to tests for homogeneity (Leveine test) and normal distribution (Kolmogorov-Smirnov test). All data were analyzed with the Student t-test, repeated measure ANOVA, or two-way ANOVA. Nested repeated measure ANOVA was used when data were collected in multiple trials in more than one session. For repeated measure ANOVA, all data were also subject to Mauchly's sphericity test, and F-ratios adjusted if violation occurred. All statistical analyses were performed using SPSS 19.0. A critical value for significance of p<0.05 was used throughout the study. Data are plotted ± SEM.

## Results

### Genetic background modifies the phenotype of mutant torsinA (Tor1a^ΔE/ΔE^) mice

To test for the presence of genetic modifiers of the ΔE-torsinA phenotype, we pursued a two-step strategy. We first intercrossed the 129-*Tor1a^ΔE/+^* mice with B6, D2 or CD1 wildtype mice. These strains were chosen because they are genetically dissimilar from each other and from 129 [21]. We then intercrossed the F1 129/“X” heterozygous offspring and assessed the phenotype of the homozygous F2 *Tor1a^ΔE/ΔE^* offspring. On average, these offspring will be 50% 129 and 50% novel strain. All strains yielded F2 progeny in the expected Mendelian ratios, indicating that D2 or CD1-ICR genes do not alter the ability of *Tor1a^ΔE/ΔE^* animals to survive through gestation and birth. The resulting litters were observed twice daily during the first two postnatal (P) days and once daily thereafter to determine the duration of survival. All pups were genotyped at death or between P2 and P3 and any remaining *Tor1a^ΔE/ΔE^* animals were monitored. *Tor1a^ΔE/ΔE^* mice on 129 (n = 21), 129/B6 (n = 26), 129/D2 (n = 53) and 129/CD1 (n = 20) backgrounds were generated and closely observed.

**Figure 1:**
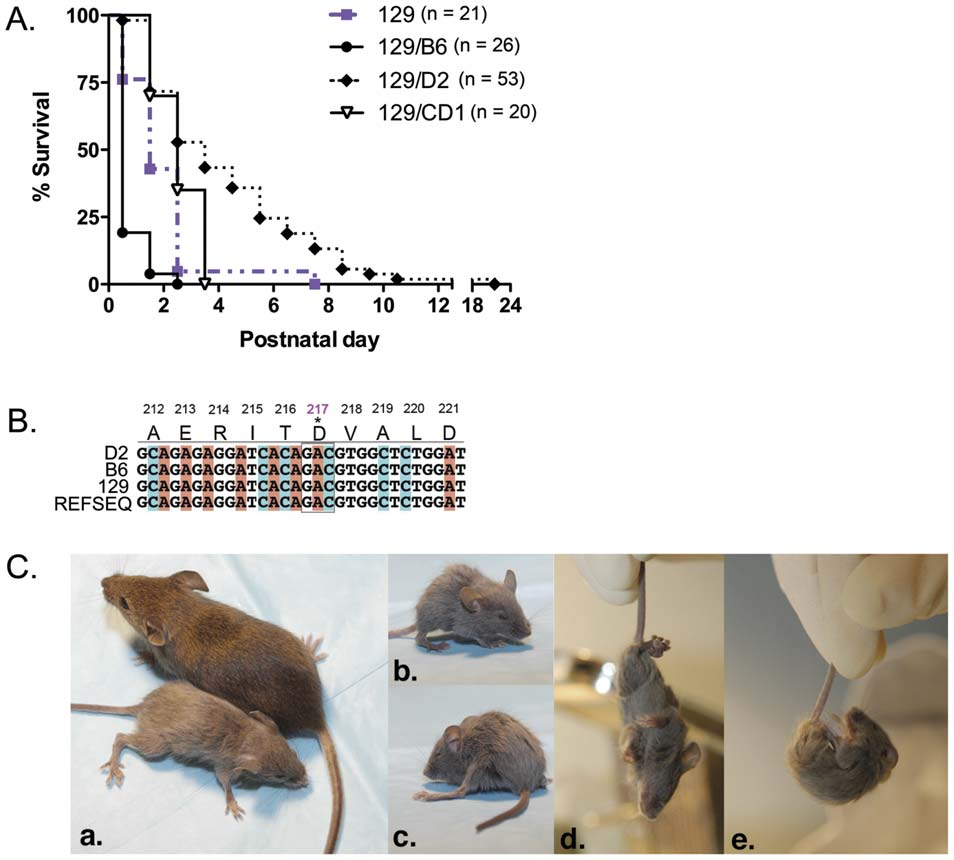
Mouse background modulates lifespan of *Tor1a^ΔE/ΔE^* mice. A. Kaplan-Meier survival curves of *Tor1a^ΔE/ΔE^* mice. D2, B6 and CD1-ICR genes modulate lifespan in *Tor1a^ΔE/ΔE^* mice compared to original 129 background (Chi-square test used to generate p values: p<0.0001 for 129 versus 129/D2, 129/C57; p<0.05 for 129 versus 129/CD1). B. CLUSTALW2 multiple sequence alignment of genomic DNA from D2, B6, and 129 wildtype mice and Refseq demonstrates that all three strains have aspartic acid (D) at position 217 (*) of the mouse torsinA protein; figure depicts base pairs which correspond to the amino acid coding sequence 212–221 (AERITDVALD) C. Long-lived F2 129/D2 *Tor1a^ΔE/ΔE^* mouse and *Tor1a^+/+^* littermate. 129/D2 *Tor1a^ΔE/ΔE^* mouse is smaller than control at postnatal day 14 (a.), poorly groomed with partially closed eyes (b.), exhibits improper limb placement (c.), and demonstrates hindlimb clasping on tail suspension tests (d., e.).

We find that each of the three background strains has a distinct effect on the ΔE-torsinA phenotype. 129/D2 *Tor1a^ΔE/ΔE^* mice live significantly longer than 129-*Tor1a^ΔE/ΔE^* mice. The D2 background significantly increases median survival to 3.5 days, compared to 1.5 days on 129 background (χ^2^(1) = 14.60; p<0.0001 (129 vs 129/D2); Figure 1A). The longest-surviving 129/D2 *Tor1a^ΔE/ΔE^* animal lived for 21 days, and 13.2% live longer than 7 days. Mice that live through the end of the first postnatal week develop abnormal motor behavior, exhibiting tremor and prolonged twisting movements during gait, particularly of the hindlimbs (Figure 1C; Video S1). To test whether we could further enhance the lifespan of 129/D2 *Tor1a^ΔE/ΔE^* mice, we continued to backcross the ΔE mutation for 10 generations onto the D2 background. However, we find that F10 D2·*Tor1a^ΔE/ΔE^* mice do not live significantly longer than mice on the 129 background (χ^2^(1) = 0.62; p = 0.43). In contrast to the D2 background, the B6 background significantly decreases median lifespan to 0.5 days (χ^2^(1) = 16.31; p<0.0001 (129 vs. 129/B6)). The CD1 background also significantly alters lifespan, increasing the median survival from 1.5 days (pure 129) to 2.5 days (χ^2^(1) = 5.207; p<0.05 (129 vs CD1/129). Unlike D2 background effect, however, all *Tor1a^ΔE/ΔE^* animals die by P3.5. These data are consistent with existence of genetic factors that modify the ΔE-torsinA related phenotypes (Figure 1A).

Interestingly, we do not find an effect of D2 background on NE bleb formation. Examination of various brain regions from E18.5 129/D2 and 129*-Tor1a^ΔE/ΔE^* embryos reveals similar percentages of NE bleb formation (129/D2% vs. 129% as follows): cortex (8% vs. 7%), striatum (6% vs. 1.2%), and cerebellum (71% vs. 81%; n = 2 for each genetic background; Figure S1).

### Gene sequencing for polymorphism (Aspartic acid/Histidine 217)

The only genetic factor linked to the penetrance of the ΔE-torsinA phenotype in humans is a coding polymorphism of torsinA itself. Penetrance for ΔE-torsinA gene carriers whose *wild type* torsinA contains a histidine (H) at position 216 is 3% compared to 35% for the more common aspartic acid (D) at the same position [22], [23]. However, this polymorphism cannot account for the effects of the D2 or B6 background, since in congenic *Tor1a^ΔE/ΔE^* mice, both *Tor1a-ΔE* alleles derive from the original 129 ES cell line used for gene targeting. It could however, account for any behavioral differences that may be observed in the heterozygous disease state *Tor1a^ΔE/+^* mice. Therefore, we sequenced the *Tor1a* allele from 129, B6 and D2 mice to determine whether there were differences at this position (217 in the mouse protein) that would inform our choice of background strain for modeling the disease in heterozygous mice. We find that all three strains carry an aspartic acid (D), preventing us from exploiting this polymorphism in this context (Figure 1B).

### B6·Tor1a^ΔE/+^ (DYT1) mice do not have baseline motor abnormalities

The contribution of genes from the B6 strain of mice significantly shortened lifespan in the resulting F2 129/B6 *Tor1a^ΔE/ΔE^* mice (compared to the pure 129 background). Therefore, we did all behavioral analysis on congenic B6·*Tor1a^ΔE/ΔE^* mice with the rationale that this more susceptible strain might reveal motor abnormalities we did not observe previously in heterozygous *Tor1a^ΔE/+^* mice on the 129 background (there are no significant differences in open field or rotarod between 129-*Tor1a^ΔE/+^* and 129-*Tor1a^+/+^* mice at 6, 9, and 12 months of age – n = 16 WT and 14 mutants; data not shown). While we realize that the decreased lifespan may be the result of an interaction between 129 and B6 genes, we chose to backcross the 129 *Tor1a^ΔE/+^*heterozygous mice to the B6 background to reduce any background noise that may occur on a mixed background. Furthermore, for all behavioral testing we only used adult mice greater than 3 months of age to ensure that any developmentally dependent phenotype would be fully manifest.

#### Open field

**Figure 2:**
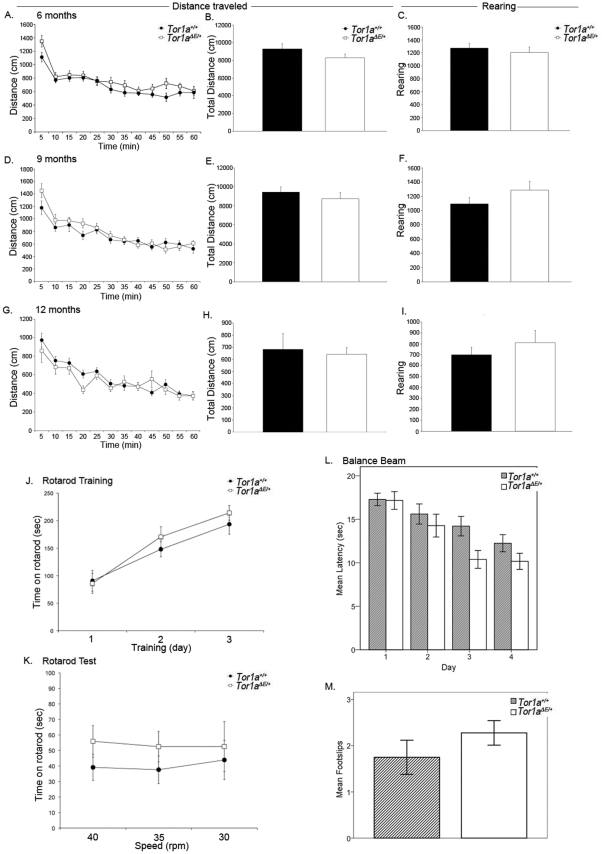
B6·*Tor1a^ΔE/+^* mice do not have baseline motor abnormalities. Male B6·*Tor1a^ΔE/+^*and B6·*Tor1a^+/+^* animals were monitored for gross motor abnormalities. Horizontal activity in the open field for 60 min (5 min per point) sessions at 6 mos (A), 9 mos (D), and 12 mos of age (G) does not differ between genotypes, rm-ANOVA, (F[11,187] = 1.266, p = 0.25 at 6 mos; F[4.67,107.5] = 1.88, p = 0.11 at 9 mos; F[5.02,115.52] = 1.56, p = 0.18 at 12 mos). Total distance traveled and total rearing over 60 min are shown as bargraphs (B, C, E, F, H, I). Each bar represents the mean of total activity over one hour. Assessment of total horizontal distance traveled and total rearing by student's T-test, also found no difference between genotypes at any of the observed ages total distance: t[17] = 1.40 ; p = 0.18, at 6 mos; t(23) = 0.84, p = 0.92, at 9 mos; t[23] = 0.65, p = 0.52, at 12 mos (B, E, H), and for total rearing: t[17] = 0.62, p = 0.54, at 6 mos; t[23] = −0.90, p = 0.38, at 9 mos; t[23] = −0.90, p = 0.38 at 12 mos (C, F, I). (J) One year old B6·*Tor1a^ΔE/+^*and B6·*Tor1a^+/+^* mice learn at the same rate during the three consecutive training days on the accelerating rotarod, rm-ANOVA, significant main effect of training day F[2, 46] = 72.06, p = 0.00 but do not perform differently (no interaction between training day and genotype: F[1.57, 46)] = 1.25, p = 0.29). (K) Both groups perform the same on the testing day (3 fixed speeds, 4 trials each), rm-ANOVA: no interaction between speed and genotype (F[1], [23] = 0.91 (p = 0.35). (L) Seven month old B6·*Tor1a^ΔE/+^*and B6·*Tor1a^+/+^* mice perform similarly on the balance beam. Latency to cross the 5 mm square beam is shown for 4 consecutive days (2 trials/day), rm-ANOVA: main effect of day: F[2.83, 63] = 10.12 (p = 0.00), no interaction between day and genotype, F[3,63] = 0.83 (p = 0.48). (M) The number of footslips is shown for the last day of testing and no difference is found, T[20.85] = 1.16; (p = 0.26).

To test motor activity in B6·*Tor1a^ΔE/+^* mice, we placed naïve male mice in the open field and measured horizontal and rearing locomotor activity over 60 minutes. This assessment was performed at 6, 9 and 12 months of age (n = 10 *Tor1a^+/+^* and 9 *Tor1a^ΔE/+^* at 6 months; n = 15 *Tor1a^+/+^* and 10 *Tor1a^ΔE/+^* mice at 9 and 12 months of age). No significant differences were observed between B6·*Tor1a^ΔE/+^* and B6·*Tor1a^+/+^* mice at any of the ages tested. All animals habituated to the open field at the same rate and performed similarly as assessed by total distance traveled and total rearing (Figure 2A–I).

#### Rotarod

To assess balance and coordination we performed rotarod testing at 12 months of age (n = 15 *Tor1a^+/+^* and 10 *Tor1a^ΔE/+^*mice). The ability to perform this task is measured by assessing the latency to fall from the rotarod (with longer latency representing improved performance). During the three-day training component of the task (2 trials per day), both groups of mice exhibited significant improvement on the accelerating rotarod, but no difference was found between the two groups, indicating that both genotypes learned the task at the same rate. Similarly, there was no significant difference in performance on the “testing day” when the animals are tested at the fixed speeds of 40 rpm, 35 rpm and 30 rpm (4 trials per speed). These data demonstrate that in the rotarod task, the ΔE mutation does not significantly impair motor learning or gross motor skills such as balance and coordination (Figure 2J–K).

#### Beam-walking test

To further assess fine motor behavior and balance in the B6·*Tor1a^ΔE/+^*mice, we used the beam-walking paradigm. A separate cohort of seven-month old B6·*Tor1a* naïve mice were trained to traverse a 5 mm square plexiglass beam on two trials for three consecutive days, and latency to cross was measured (with longer latency indicating impaired performance). Latency to cross was measured for the training and testing days, and on the fourth “testing day” we also quantified the number of foot-slips. We noted a significant improvement in the time it took for mice to traverse the beam (one-way rm-ANOVA, main effect of day: F[2.83, 63] = 10.12, p = 0.00) however, no significant difference was observed for latency between wild type (n = 14) and mutant mice (n = 9) during training or on the testing day. Similar to the findings for latency, no significant difference was observed between wild type and mutant mice for foot-slips (Figure 2L–M).

### B6·Tor1a^ΔE/+^ (DYT1) mice do not display altered responses to drug challenges

Anti-muscarinic drugs can be effective in treating the symptoms of DYT1 dystonia and DYT1-torsinA transgenic mice are reported to show an abnormal interplay between the dopaminergic and cholinergic systems in electrophysiological studies [24], [25], [26]. To probe these neurochemical systems, we challenged *Tor1a^+/+^* and *Tor1a^ΔE/+^*mice with the muscarinic receptor antagonist, scopolamine, and the dopamine transporter reuptake inhibitor, GBR12909, measuring the behavioral response to these drugs in the open field.

#### Scopolamine

**Figure 3:**
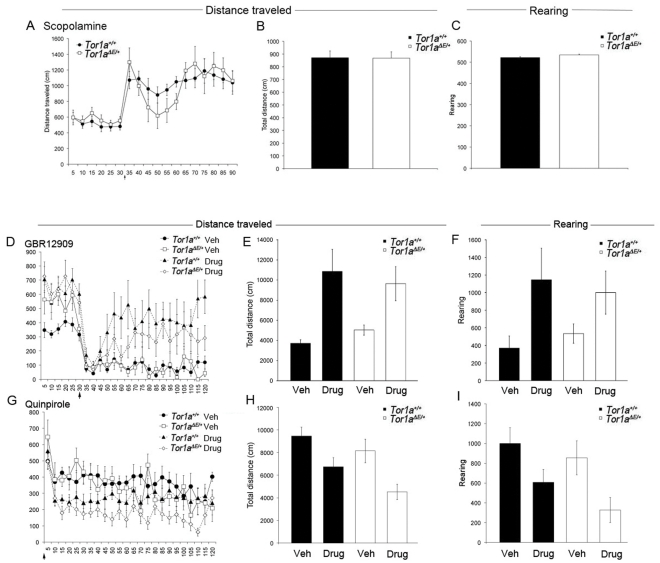
B6·*Tor1a^ΔE/+^* mice do not respond differently to pharmacological challenges in the open-field. Locomotor activity of B6·*Tor1a^ΔE/+^* and B6·*Tor1a^+/+^* mice was monitored for 90 minutes (5 min per point) total. After a 30 minute habituation period, mice were injected with (A) scopolamine (1.0 mg/kg) or (D) GBR12909 (5.0 mg/kg) or vehicle (indicated by arrow) and monitored for an additional 60 minutes. Scopolamine and GBR12909 stimulated locomotor activity of both genotypes but there was no interaction between genotype and drug. Scopolamine challenge, rm-ANOVA reveals no main effect of genotype and no significant interaction between time and genotype (F[2.52, 55.35] = 1.05, p = 0.37) of horizonal activity in the openfield (A). When totaled over the entire time in the openfield, there is no significant effect of genotype on total horizontal distance, t[22] = 0.040, (p = 0.97) or total rearing t[22)] = −0.143, (p = 0.86; B–C). GBR challenge, rm-ANOVA demonstrated a significant main effect of drug, F[1], [25] = 7.39, p = 0.01, but no significant main effect of genotype (F[1], [25] = .287, p = 0.60) and no significant interaction between drug and genotype (F[1], [25] = 0.08, p = 0.78; D). Evaluation of total horizontal distance and total rearing with two-way ANOVA finds significant main effect of drug (F([1], [20] = 16.02, p = 0.00 (total horizontal distance); F[1], [21] = 0.20, p = 0.66 (total rearing)) but no interaction between drug and genotype (F[1], [20] = 1.46, p = 0.24 (total horizontal distance); F[1], [21] = 0.50, p = 0.49 (total rearing; E–F). (G) Quinpirole (0.1 mg/kg) was injected at the start of the open field session and locomotor activity was monitored for 120 minutes. Quinpirole inhibited locomotor activity of both groups of mice compared to vehicle but did not elicit a significant difference between genotypes. Rm-anova demonstrated a significant main effect of drug F[1], [29] = 13.65, p = 0.001, a significant main effect of genotype (F[1], [29] = 0.28, p = 0.05), but no significant interaction between drug and genotype (F[1], [29] = 0.28, p = 0.06). (H–I) Examination of total horizontal distance and total rearing with two-way ANOVA finds a significant main effect of drug (F[1], [30] = 11.330, p = 0.002 (total horizontal distance); F[1], [30] = 9.68, p = 0.004 (total rearing)) but no interaction between drug and genotype (F[1], [30] = 0.74. p = 0.40 (total horizontal distance); F[1], [30] = 0.20, p = 0.66 (total rearing).

Following a 30-minute habituation period in the open field, we find no significant difference in the ability of scopolamine (1.0 mg/kg) to stimulate either horizontal or rearing locomotor behavior in B6·*Tor1a^ΔE/+^*or B6·*Tor1a^+/+^* mice (n = 14 *Tor1a^+/+^* and 10 *Tor1a^ΔE/+^*mice; Figure 3A–C).

#### GBR12909

Similar to our results with scopolamine, following habituation we find no significant difference in the ability of GRB12909 (5.0 mg/kg, i.p.) to alter horizontal/rearing behavior in B6·*Tor1a^ΔE/+^*or B6·*Tor1a^+/+^* mice (n = 5 *Tor1a^+/+^* and 8 *Tor1a^ΔE/+^* mice for drug group and 7 *Tor1a^+/+^* and 4 *Tor1a^ΔE/+^* mice for vehicle groups; Figure 3D–F).

#### Quinpirole

Since dopaminergic pathophysiological effects described in transgenic animals have been attributed to D2 dopamine receptor function, we also challenged mice with quinpirole (0.1 mg/kg, i.p.), a selective D2 dopamine receptor antagonist. We find no difference in the effect of quinpirole on horizontal/rearing locomotor activity in B6·*Tor1a^ΔE/+^*or B6·*Tor1a^+/+^*mice (n = 10 *Tor1a^+/+^* and 11 *Tor1a^ΔE/+^*mice for drug groups, 7 *Tor1a^+/+^* and 6 *Tor1a^ΔE/+^*mice for vehicle groups; Figure 3G–I).

## Discussion

Our study is the first to investigate the effects of genetic background on the phenotype of torsinA mutant mice, a feature of particular interest because of the reduced penetrance of the disease, some of which may relate to genetic modifiers in the human population. We identified background strains that are able to suppress (D2 and CD1) or enhance (B6) the lethality of 129-*Tor1a^ΔE/ΔE^* mice. These genetic backgrounds produced survival times ranging from less than 12 hours to up to 3 weeks, depending on the parental strains used for the F2 intercross (Figure 1). Future mapping of the genes responsible for these effects may provide insight into the torsinA pathway, which remains poorly understood. Alternatively, it is possible that these variants alter lifespan independently of the torsinA pathway, for example by making the pups more (or less) able to withstand the effects of torsinA dysfunction.

A barrier to progress in dystonia research is the lack of an animal model with overt abnormal movements, and a key aim of our experiments was to identify background strains that might enable us to develop such a model. Strikingly, the long-lived D2·*Tor1a^ΔE/ΔE^* pups exhibited a noticeable tremor, abnormal limb placement and limb weakness, and a delayed righting reflex (Video S1). While this abnormal motor behavior likely results from torsinA-related neural dysfunction, these pups do not feed well and appear generally ill, a confounding factor that complicates the interpretation of this phenotype. This finding was nevertheless encouraging, and we tried to build on it to create healthy mice that display abnormal motor function (like the disease). Since an increase from one to two mutated torsinA alleles causes early lethality in the majority of mice, we tried to temper this phenotype by further backcrossing to the apparently more permissive D2 background and analyzing D2·*Tor1a^ΔE/ΔE^* mice. The variability and enhanced lifespan diminished when *Tor1a^ΔE/+^* animals were backcrossed more than 10 generations to a congenic D2 background. In fact, the lifespan of *Tor1a^ΔE/ΔE^* on the D2 background was indistinguishable from those on the original 129 background. These findings indicate that the extended lifespan of F2 129/D2 mice was due to a combination of homozygous and heterozygous allelic effects. The debilitated nature of these mice precluded further study of these animals.

We find that 129-*Tor1a^ΔE/+^* mice have no apparent behavioral abnormalities. Because the B6 background significantly decreased the survival of torsinA mutant mice, we explored whether this background would enable us to detect phenotypic effects of the ΔE mutation in the heterozygous animals. We generated congenic B6·*Tor1a^ΔE/+^* mice and tested them in several behavior paradigms: open field (with and without pharmacological challenge), rotarod, and balance beam. Similar to the 129S6/SvEvTac background, however, there were no significant differences between *Tor1a^ΔE/+^* and *Tor1a^+/+^* on the B6 background.

A previous study of *Tor1a^ΔE/+^* mice on a mixed 129/B6 background reported hyperactivity (significantly increased distance traveled) in a 10-minute open field test and normal rearing activity [15]. Interestingly, we find a trend in the opposite direction, with B6·*Tor1a^ΔE/+^* mice appearing hypoactive during the first 5 minutes of the open field test, (p = 0.05). *Dang et. al.* also measured behavior on the rotarod and in the beam walking test. They report that wild type and mutant mice perform equally well on the rotarod, however mutant mice show significantly more footslips than controls on the balance beam test. There was no difference in latency to cross the beam, however. There were some differences in the execution of experiments between our studies presented here and those of *Dang et al.* First, we examined spontaneous locomotor activity in the open field in 5-minute bins for 60 minutes, while *Dang et al.* examined only the first 10 minutes. Second, *Dang et al.* examined balance and coordination with several different sized beams on the beam walking test (both square and round beams ranging in size from 17 mm diameter/width to 7 mm width, while we trained and tested mice on a more difficult 5 mm width square beam. These data, and our finding of normal behavior in the open field and beam walking suggest that ΔE-torsinA may cause subtle behavioral abnormalities. However, it is also possible that the confounding effects of mixed background are responsible for the subtle abnormalities identified by *Dang et al*.

There are limitations of genetic backcrossing that may be relevant to studies of torsinA. Although nearly all loci become homogenous by approximately the tenth generation of backcrossing, the mutated allele and closely linked flanking sequence from the original background persist. With each successive backcross the flanking sequence surrounding the gene of interest shortens, but frequently several MB of the original background remain. TorsinB (a close homolog of torsinA) is located adjacent to torsinA, so all of the backgrounds tested almost certainly carry the original 129-torsinB allele. A previous study demonstrated that torsinA and torsinB share redundant functions in multiple cell types, raising the possibility that torsinB influences disease penetrance [9]. This may explain, at least in part, the absence of a behavioral phenotype in *Tor1a^ΔE/+^* mice.

Similar considerations pertain to the one reported genetic modifier of disease penetrance in DYT1 dystonia. A non-synonymous SNP in the coding sequence for residue 216 encodes aspartic acid (D) in 88% and histidine (H) in 12% of control population alleles, and the D216H allele is reported to significantly reduce disease penetrance when present in the normal allele in *trans* to the mutated allele [23], [27]. We found the “D” allele at the analogous murine residue (217) in all of the strains used in this study, further demonstrating the highly conserved features of the *Tor1a* gene and indicating that this SNP does not affect penetrance in *Tor1a^ΔE/+^* mice. Several other SNPs are present in the 5′ and 3′ UTR regions of TOR1A, as well as a single-base-pair deletion in the 3′ UTR (G-del), but these features are not known to be associated with the penetrance of DYT1 dystonia. On the other hand, several SNPs in the 3′ UTR may influence the onset and propensity to spread in adult-onset primary dystonia [28], [29].

Etiological animal models (i.e., those based on known causes of human disease) may offer insight into disease pathogenesis even if they do not replicate the outward symptoms of the disease. In fact, diffusion tensor imaging (DTI) of B6·*Tor1a^ΔE/+^*mice demonstrates microstructural abnormalities in the cerebellothalamocortical and thalamocortical tracts [22] similar to those observed in non-penetrant DYT1 carriers [6], [30], [31] indicating that these mice model non-manifesting carriers. Thus, while genetic modifiers seem likely to account for at least part of variable penetrance and expressivity of the ΔE-*Tor1a* allele, additional factors may be required to convert *Tor1a^ΔE/+^*mice (or patients) from non-manifesting to overt disease. For example, stress exposure or excessive motor activity (e.g., prolonged wheel running) may be required “second hits”. Future studies aimed at identifying such factors will therefore be required to generate torsinA mutant mice that exhibit abnormal movements, a critically needed reagent if we are to use animal models to dissect the neurobiological substrates of dystonic movements.

## Supporting Information

Figure S1 ***Tor1a^ΔE/ΔE^*** **mice exhibit similar neuronal NE blebbing ultrastructure on the 129 and 129/D2 background at age E18.5.** Nuclear envelope abnormalities previously described are apparent at E18.5 in cortex of *Tor1a^ΔE/ΔE^* mice when viewed by electron microscopy. A. Normal E18.5 cortical neuronal nuclear envelope. B. Abnormal NE with bleb visible between inner and outer nuclear membrane in 129-*Tor1a^ΔE/ΔE^* mouse cortical neuron. C. Abnormal NE with bleb visible between inner and outer nuclear membrane in 129/D2·*Tor1a^ΔE/ΔE^* mouse cortical neuron. Scale bars, 500 nm. N, nucleus; C, cytosol.
(TIF)

Video S1 **Long-lived postnatal day 8 129/D2** ***Tor1a^ΔE/ΔE^*** **mouse and littermate.** 129/D2 *Tor1a^ΔE/ΔE^* mice are strikingly smaller compared to littermate controls and exhibit obvious motor dysfunction, including tremor, improper limb placement, and limited balance.
(MOV)

## References

[1] Tanabe LM, Kim CE, Alagem N, Dauer WT (2009). Primary dystonia: molecules and mechanisms.. Nat Rev Neurol.

[2] Ozelius L, Kramer PL, Moskowitz CB, Kwiatkowski DJ, Brin MF (1989). Human gene for torsion dystonia located on chromosome 9q32–q34.. Neuron.

[3] Ozelius LJ, Page CE, Klein C, Hewett JW, Mineta M (1999). The TOR1A (DYT1) gene family and its role in early onset torsion dystonia.. Genomics.

[4] Eidelberg D, Moeller JR, Antonini A, Kazumata K, Nakamura T (1998). Functional brain networks in DYT1 dystonia.. Ann Neurol.

[5] Trost M, Carbon M, Edwards C, Ma Y, Raymond D (2002). Primary dystonia: is abnormal functional brain architecture linked to genotype?. Ann Neurol.

[6] Argyelan M, Carbon M, Niethammer M, Ulug AM, Voss HU (2009). Cerebellothalamocortical connectivity regulates penetrance in dystonia.. J Neurosci.

[7] Niethammer M, Carbon M, Argyelan M, Eidelberg D (2011). Hereditary dystonia as a neurodevelopmental circuit disorder: Evidence from neuroimaging.. Neurobiol Dis.

[8] Nadeau JH (2005). Listening to genetic background noise.. N Engl J Med.

[9] Kim CE, Perez A, Perkins G, Ellisman MH, Dauer WT (2010). A molecular mechanism underlying the neural-specific defect in torsinA mutant mice.. Proc Natl Acad Sci U S A.

[10] Goodchild RE, Dauer WT (2005). The AAA+ protein torsinA interacts with a conserved domain present in LAP1 and a novel ER protein.. J Cell Biol.

[11] Giles LM, Li L, Chin LS (2009). Printor, a novel torsinA-interacting protein implicated in dystonia pathogenesis.. J Biol Chem.

[12] Sharma N, Baxter MG, Petravicz J, Bragg DC, Schienda A (2005). Impaired motor learning in mice expressing torsinA with the DYT1 dystonia mutation.. J Neurosci.

[13] Shashidharan P, Sandu D, Potla U, Armata IA, Walker RH (2005). Transgenic mouse model of early-onset DYT1 dystonia.. Hum Mol Genet.

[14] Grundmann K, Reischmann B, Vanhoutte G, Hubener J, Teismann P (2007). Overexpression of human wildtype torsinA and human DeltaGAG torsinA in a transgenic mouse model causes phenotypic abnormalities.. Neurobiol Dis.

[15] Dang MT, Yokoi F, McNaught KS, Jengelley TA, Jackson T (2005). Generation and characterization of Dyt1 DeltaGAG knock-in mouse as a model for early-onset dystonia.. Exp Neurol.

[16] Goodchild RE, Kim CE, Dauer WT (2005). Loss of the dystonia-associated protein torsinA selectively disrupts the neuronal nuclear envelope.. Neuron.

[17] Cookson MR, Clarimon J (2005). Dystonia and the nuclear envelope.. Neuron.

[18] Crawley JN, Belknap JK, Collins A, Crabbe JC, Frankel W (1997). Behavioral phenotypes of inbred mouse strains: implications and recommendations for molecular studies.. Psychopharmacology (Berl).

[19] Kearney JA (2011). Genetic modifiers of neurological disease.. Curr Opin Genet Dev.

[20] Shakkottai VG, do Carmo Costa M, Dell'Orco JM, Sankaranarayanan A, Wulff H (2011). Early changes in cerebellar physiology accompany motor dysfunction in the polyglutamine disease spinocerebellar ataxia type 3.. J Neurosci.

[21] Beck JA, Lloyd S, Hafezparast M, Lennon-Pierce M, Eppig JT (2000). Genealogies of mouse inbred strains.. Nat Genet.

[22] Bressman SB (2007). Genetics of dystonia: an overview.. Parkinsonism Relat Disord.

[23] Risch NJ, Bressman SB, Senthil G, Ozelius LJ (2007). Intragenic Cis and Trans modification of genetic susceptibility in DYT1 torsion dystonia.. Am J Hum Genet.

[24] Pisani A, Martella G, Tscherter A, Bonsi P, Sharma N (2006). Altered responses to dopaminergic D2 receptor activation and N-type calcium currents in striatal cholinergic interneurons in a mouse model of DYT1 dystonia.. Neurobiol Dis.

[25] Martella G, Tassone A, Sciamanna G, Platania P, Cuomo D (2009). Impairment of bidirectional synaptic plasticity in the striatum of a mouse model of DYT1 dystonia: role of endogenous acetylcholine.. Brain.

[26] Napolitano F, Bonito-Oliva A, Federici M, Carta M, Errico F (2010). Role of aberrant striatal dopamine D1 receptor/cAMP/protein kinase A/DARPP32 signaling in the paradoxical calming effect of amphetamine.. J Neurosci.

[27] Leung JC, Klein C, Friedman J, Vieregge P, Jacobs H (2001). Novel mutation in the TOR1A (DYT1) gene in atypical early onset dystonia and polymorphisms in dystonia and early onset parkinsonism.. Neurogenetics.

[28] Clarimon J, Asgeirsson H, Singleton A, Jakobsson F, Hjaltason H (2005). Torsin A haplotype predisposes to idiopathic dystonia.. Ann Neurol.

[29] Kamm C, Asmus F, Mueller J, Mayer P, Sharma M (2006). Strong genetic evidence for association of TOR1A/TOR1B with idiopathic dystonia.. Neurology.

[30] Ulug AM, Vo A, Argyelan M, Tanabe L, Schiffer WK (2011). Cerebellothalamocortical pathway abnormalities in torsinA DYT1 knock-in mice.. Proc Natl Acad Sci U S A.

[31] Carbon M, Su S, Dhawan V, Raymond D, Bressman S (2004). Regional metabolism in primary torsion dystonia: effects of penetrance and genotype.. Neurology.

