# Supplementary figures and images for: Genetic Background Modulates the Phenotype of a Mouse Model of DYT1 Dystonia
Source: PLoS One. 2012 Feb 29;7(2):e32245. doi: 10.1371/journal.pone.0032245 (PMC3290549; doi:10.1371/journal.pone.0032245)

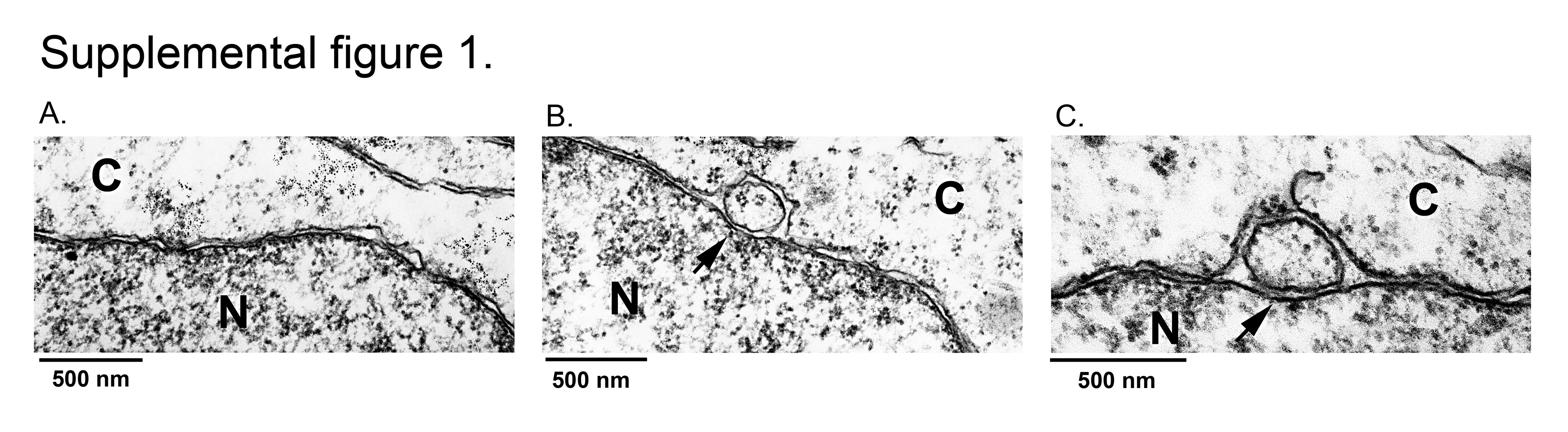

Supplement: Figure S1 — Tor1aΔE/ΔE mice exhibit similar neuronal NE blebbing ultrastructure on the 129 and 129/D2 background at age E18.5. Nuclear envelope abnormalities previously described are apparent at E18.5 in cortex of Tor1aΔE/ΔE mice when viewed by electron microscopy. A. Normal E18.5 cortical neuronal nuclear envelope. B. Abnormal NE with bleb visible between inner and outer nuclear membrane in 129-Tor1aΔE/ΔE mouse cortical neuron. C. Abnormal NE with bleb visible between inner and outer nuclear membrane in 129/D2·Tor1aΔE/ΔE mouse cortical neuron. Scale bars, 500 nm. N, nucleus; C, cytosol. (TIF) [file pone.0032245.s001.tif]
